# Effect of a mechanical grooming brush on the behavior and health of recently weaned heifer calves

**DOI:** 10.1186/s12917-019-2033-3

**Published:** 2019-08-08

**Authors:** Ana Velasquez-Munoz, Diego Manriquez, Sushil Paudyal, Gilberto Solano, Hyungchul Han, Robert Callan, Juan Velez, Pablo Pinedo

**Affiliations:** 1 Department of Animal Sciences, Colorado State University, Fort Collins, CO 80523 USA; 2 Department of Animal and Veterinary Science, California Polytechnic University Pomona, Pomona, CA 91768 USA; 3 Department of Clinical Sciences, College of Veterinary Medicine and Biomedical Sciences, Colorado State University, Fort Collins, CO 80521 USA; 4 Aurora Organic Farms, Boulder, CO 80302 USA

**Keywords:** Mechanical brush, Welfare, Behavior, Dairy heifer

## Abstract

**Background:**

Calf stress at weaning and during transition to group pens represents a concern in dairy operations. Favoring natural behaviors, such as grooming, may help on reducing this challenge. Our objective was to evaluate the effect of a mechanical grooming brush on behavior and health of recently weaned calves, after transferring from individual to group housing. Two treatment groups (control [CON, *n* = 81]; automated brush [AB, n = 81]) were compared enrolling Holstein heifers (94 ± 7 d old) that were monitored for 20 days. Four cohorts, considering one CON and one AB group (19–20 calves/pen/cohort) were enrolled sequentially. Each calf was weighed, clinically evaluated, and affixed with a 3-D accelerometer sensor attached to the ear at enrolment. Continuous measurements (min/h) were generated for the following behaviors: Not-active, active, highly active, eating, and ruminating. Cameras for continuous video recording were installed in each pen, and calves were weighted at the last day of the study (d 20). Behavioral data were summarized as daily averages (min/h). Data was examined using repeated measures analysis for nested factors, with day as the time unit.

**Results:**

Overall, calves had their first interaction with the brush within 2.5 days with a mean (SE) of 7 (±9.6) h after being transferred to group pens. A significant effect was determined for the interaction day by treatment on the time spent not-active and eating. Average not-active time was greater in CON compared to AB (22.8 ± 0.82 min/h vs. 21.7 ± 0.82 min/h), while eating time was greater in AB compared with CON (7.01 ± 0.40 min/h vs. 6.43 ± 0.40 min/h). Treatment groups had a similar weight gain and time to the first disease.

**Conclusions:**

We concluded that the use of a mechanical brush influenced behavior, reducing not-active time, while increasing eating time. The consequences of this change in activity require further investigation.

## Background

The weaning process is characterized not only by a change in diet, but also for a new social and physical environment that calves might adapt to in a short period of time [1]. The success of recently weaned calves on overcoming these challenges is bonded with the characteristics of their new housing system, including space per animal, access to food and water, and shelter availability [2].

Grooming is an innate behavior that might help cattle to cope with stress [3]. At the same time, grooming has been described as an activity that is compromised during disease [4, 5]. In the recent two decades, different grooming devices have been developed and the number of farms providing automated brushes to lactating cows has increased [6], with the idea of improving performance through access to enriched environments [7]. In addition, brushes specifically designed for young calves are gaining acceptance and might represent a tool to help calves to better adapt to changes during the transitioning period after weaning.

Enriched environments for young animals raised in confinement have a positive effect on the time animals engage in locomotor play, as described for dairy calves raised in hutches furnished with a stationary brush, a rubber chain, and a calf “lollie” [8]. Additionally, research in pigs has reported greater growth rates, increased feed intake, increased exploratory behavior, and reduced inactive and aggressive time [9]. Dairy calves experience stress in multiple stages of their life. Therefore, allowing natural behaviors through an enriched environment might influence their behavior, and it might be a tool to assess early stages of disease and discomfort, as it has been reported in adult cattle [2, 4, 5, 10]. In addition, research performed in dairy calves has demonstrated a high acceptance to these tools and an increase in self-grooming [8, 11–13].

Changes in behavior, health status, and performance are parameters considered when assessing the effectiveness of different management decisions or environment improvements [14, 15]. Therefore, the use of accelerometer sensors or devices that accurately measure behavior opens new opportunities for research [16–19]. We hypothesized that the addition of a grooming device in group pens of weaned calves would have an effect in core behaviors (eating, rumination, resting and active time) and would delay and reduce the presentation of clinical disease. Therefore, the specific objective of this study was to evaluate the effect of an automated grooming brush on the behavior and health of recently weaned calves transitioning from individual to group housing.

## Results

### Health status

During the complete study period, 29 (18%) calves were treated for respiratory disease (CON = 19, AB = 10; Table 1) and 25 animals left the study due to disease, as they were moved to a smaller pen for closer observation and care (CON = 16, AB = 9, Table 1).

**Table 1:** Frequency of calves receiving medical treatment (*P* = 0.06) and leaving the study due to disease (*P* = 0.12) by enrollment group during the 20 days in group housing

	Enrollment group				
Group	1	2	3	4	Total
Calves treated					
Control, n (%)	6 (21)	4(14)	5 (17)	4 (14)	19 (66)
Automated brush, n (%)	5 (17)	2 (7)	3 (10)	0 (0)	10 (34)
Total, n (%)	11 (38)	6 (21)	8 (27)	4 (14)	29 (100)
Calves leaving the pen					
Control, n (%)	5 (20)	4 (16)	4 (16)	3 (12)	16 (64)
Automated brush, n (%)	5 (20)	1 (4)	3 (12)	0 (0)	9 (36)
Total, n (%)	10 (40)	5 (20)	7 (28)	3 (12)	25 (100)

**Fig. 1:**
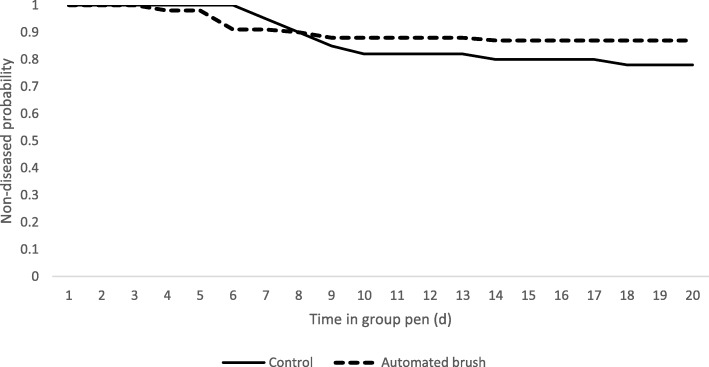
Survival curves for the time to the first disease (*P* = 0.12) by treatment group^1^.^1^Control group (solid line) and automated brush group (dashed line). Mean (SE) for days to first disease were control (CON) = 9 (0.89) d and automatic brush (AB) = 6 (0.75) d (*P* = 0.01). Adjusted hazard ratio = 1.97 (AB vs. CON treatment, *P* = 0.09)

Although the statistical analyses indicated that overall disease presentation was not associated with enrollment group (EG), control calves from EG1 presented the greatest number of sick animals. On the other hand, AB calves from EG4 did not present any disease event. A tendency for significance was determined (*P* = 0.06) for the odds of sicknesses (OR = 2.17, 95% CI = 0.95–5.18) in CON calves compared with AB calves (Table 2). No differences by treatment group (TG) were found for the time to the first disease (*P* = 0.12; Fig. 1) in the time to event analyses, although the mean (SE) time to the first disease provided by ANOVA was different for the two TG (AB = 6 [0.75] days vs. CON = 9 [0.89] days; *P =* 0.01).

**Table 2:** Results for the logistic regression analysis for calves receiving medical treatment and for calves removed due to disease by treatment group (CON = control; AB = automated brush) and enrollment group (EG)

Group	Odds Ratio	95% CI	*P*-Value
Receiving treatment^a^			
CON vs. AB	2.2	0.94–5.03	0.06
EG1 vs. EG4	3.01	0.86–10.53	0.13
EG2 vs. EG4	1.63	0.41–6-39	0.77
EG3 vs. EG4	2.24	0.61–8.27	0.56
Removal due to disease^a^			
CON vs. AB	2.01	0.82–4.92	0.12
EG1 vs. EG4	3.62	0.91–14.42	0.09
EG2 vs. EG4	1.8	0.29–8.21	0.77
EG3 vs. EG4	2.59	0.61–10.92	0.51

^a^All the potential EG combinations not included in the table indicated non-significant effect for group

### Time budget by treatment group

**Fig. 2:**
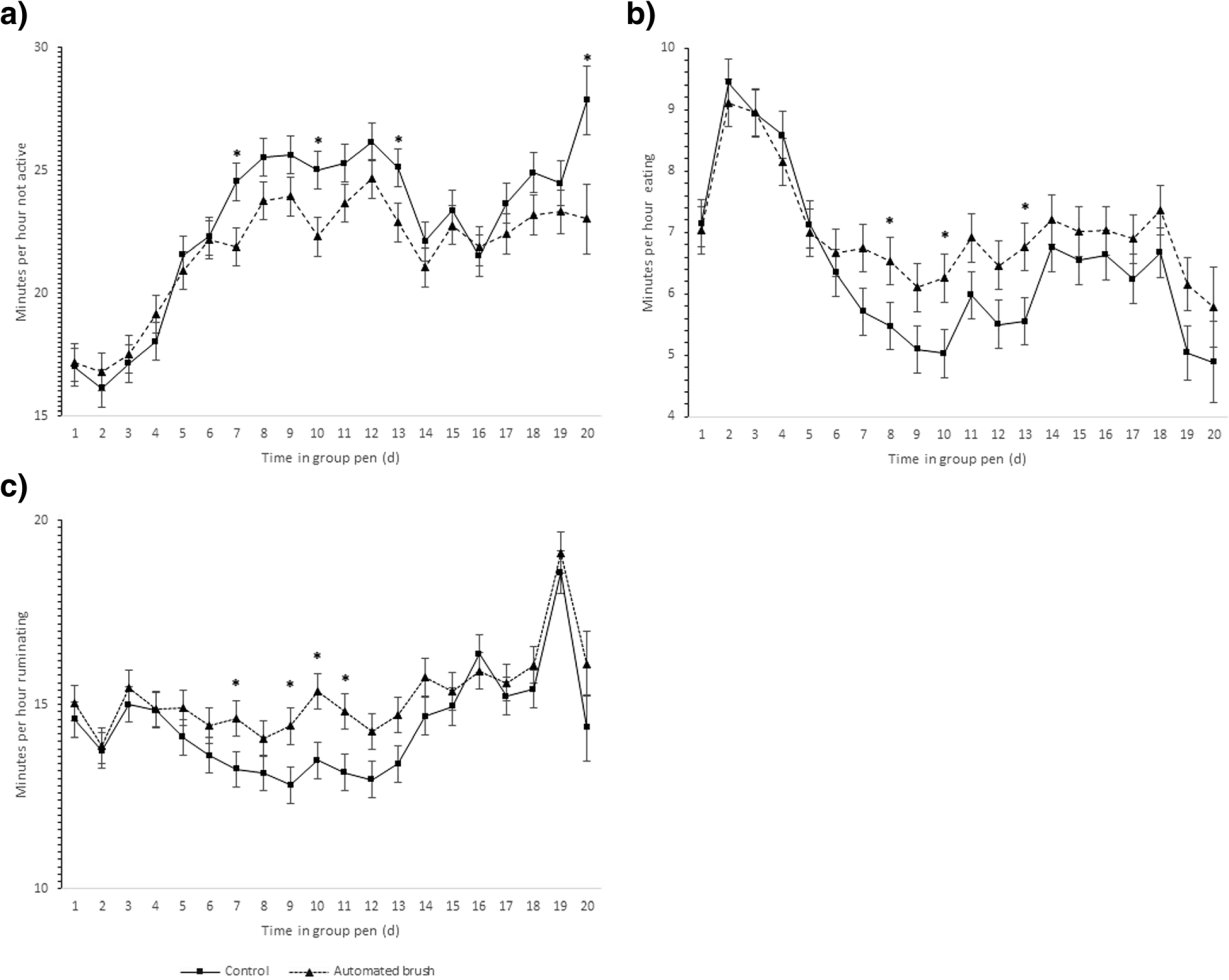
Average (SE) not-active^1^ (**a**), eating^2^ (**b**), and ruminating^3^ time (**c**; min/h) by treatment group^4^. ^1^ Control vs automated brush group *P* = 0.014. ^2^ Control vs automated brush group *P* = 0.012. ^3^ Control vs automated brush group *P* = 0.28. ^4^Control group (solid line) and automated brush (dotted line). Asterisks indicate significant difference between treatment groups

The repeated measures analyses for nested factors indicated a significant effect for the interaction day x TG on the variables not-active time and eating time. Control calves spent more time not-active in comparison with AB calves (22.8 ± 0.82 min/h vs. 21.7 ± 0.82 min/h; *P* = 0.014, Fig. 2a). Additionally, CON calves spent less time eating (6.43 ± 0.40 min/h vs. 7.01 ± 0.40 min/h; *P* = 0.012, Fig. 2b). No differences were found (*P* ≥ 0.05) for the interaction TG x day for active time (AB = 5.74 ± 0.31 min/h vs. CON = 6.28 ± 0.31 min/h), high active time (AB = 10.5 ± 0.38 min/h vs. CON = 10.3 ± 0.38 min/h) and ruminating time (AB = 15.24 ± 0.51 min/h vs. CON = 14.38 ± 0.52 min/h). However, the analyses indicated a significant difference in favor of AB calves for ruminating time at specific time points (d 7, 9, 10, and 11 in study; Fig. 2c).

### Weight gain and brush use

Average initial and final weights did not differ between treatment groups (103.3 kg vs. 104.0 kg and 125.3 kg vs. 127.1 for CON and AB, respectively). Consequently, calf average daily gain (ADG) was similar for both TG (CON = 0.75 ± 0.03 kg/d vs. AB = 0.77 ± 0.03 kg/d; *P* = 0.69).

As determined through video recording, 97% of calves interacted with the brush within 2.5 days. Overall, 57% of calves spent ≥1 min in the first contact with the brush. Furthermore, the use of the brush was consistent through night during the study period.

## Discussion

Our study centered on the effect of an automated grooming device on behavior and health recently weaned calves transitioning from individual to group housing.

### Health status

The weaning process and the change to group housing is a stressful period that can lead to disease [20–22]. Respiratory problems have been reported the most common disorders in weaned calves, with a reported morbidity of 11.2% [23], accounting for the 46.5% of death losses [24]. It is plausible to suggest that the provision of enrichment devices may reduce the level of boredom and stress when animals are housed in intensive productive systems [6, 14, 25], leading to an improved health. In our study, 18% of the enrolled calves exhibited clinical respiratory disease and most sick calves were identified between 5 and 13 d after transferring to group housing. A finding from the survival analysis (Fig. 1) was that after day 12 post transferring, no AB calves were detected sick, but CON calves presented respiratory disease throughout the 20 days in study (Fig. 1). The mean time to a first disease diagnosis was 6 days for CON calves and 9 for AB calves. However, the survival analyses indicated no significant effect for TG on the time to this event.

### Time budget by treatment group

Relevant to this research, the 3D accelerometer sensors used here provided continuous data and were previously validated for use in dairy calves on rumination, eating, and activity behaviors [18]. In addition, the raw data originated by ear tag sensors was representative of animal movement, and calf behavior [19]. Before the beginning of this study, a trial was conducted to assess possible problems related to the positioning of the sensor in the ear and the risk of irritation or infection of the area [18]. Some problems following ear tag application, such as infection and subsequent increased ear movement, were detected. Subsequently, as we replaced the farm ID tag with the sensor in the middle of the ear, no problems were observed in terms of lost devices, irritated ears, or bad positioning of the sensor weighting the ear down. In the aim of checking consistency of the 3D accelerometer sensors, activities performed by weaned calves were monitored during the trial and they consistently coincided with scheduled management at the farm, as observed in the pen visits and video recording.

Overall, the time budget of calves was affected by the presence of the AB. Sensor data analyzed by day suggested that AB calves spent more minutes per hour eating and less not-active time, compared with CON calves and these group differences were more evident from d 7 to d 20 in study. As expected, the behavior of calves changed during the first 7 days after transferring to group housing. The main change across time was observed in the categories high active (not significantly different between TG, data not shown) and not-active time. The patterns observed in the curves were opposite and complementary to each other. High active time averaged 15 ± 0.35 min/h for both TG at day 1 (the highest value for the study period) and decreased slowly to 10.5 ± 0.35 min/h at day 7. Contrary, not active time was at its lower level the first 7 days after transferring the calves to group housing. This pattern may reflect the change on the level of restriction of movement from individual to group housing, where open space is available, and calves gain the ability to interact with each other for the first time and explore a new environment. Similarly, eating time fluctuated in the first 7 days after transferring, with both groups evidencing a peak at day 2, probably due to a training in the feed bunk as part of the farm protocol. The described patterns for the behavior variables indicated that the study calves required one week for adaptation to this transitioning period. Interestingly, our results shown a difference in the rumination time only for days 7, 9, 10 and 11 after transferring to collective housing. Calves in AB spent more time ruminating than CON calves (extra 1.5 to 2 min/h; Fig. 2c). This finding might be related with the start of detection and treatment of respiratory diseases. Through this period, more CON than AB calves were detected with clinical signs of disease. At commingling, calves are still experiencing the weaning distress and the group housing may lead to competition for resources and hierarchy [1, 26, 27]. Consequently, this adaptation period requires special attention on detecting disease and to re-organize calves’ groups when needed.

Our results indicated that CON and AB calves spent in average 14 ± 0.52 min/h and 15 ± 0.52 min/h ruminating and 6.43 ± 0.40 min/h and 7.01 ± 0.40 min/h eating, respectively, which is within the range of previous reports, where an average of 13 min/ h for calves 95 d old and 15 min/h for calves 185 d old was determined [17].

### Weight gain and brush use

Consistent with previous studies analyzing the use of enrichment strategies in calves [8], our results indicated no effect of brush on body weight gain. Interestingly, AB calves spent more time eating but this difference did not result in greater ADG. A potential explanation for this finding may be that, in our study, calves spent only 20 days in the group pens and this time might be insufficient to assess differences in ADG, especially if a transitional period is included in the total exposure time. In addition, AB calves showed less not-active time, which may have resulted in usage of energy resources.

The video recording evidenced that 97% of the calves in the study interacted with the brush in a lapse of 2.5 days. In agreement, a published report indicated that beef calves 7 to 9-month-old made at least 1 contact with a brush in a period of 5 days [12]. Similarly, in a different study, 98% of 72 dairy calves that were monitored from day 40 and 70 to day 82 to 98 of life interacted with a brush, when evaluated at 3 time points [11].

In addition, it has been described that 57 and 79% of adult cows used the brush within the first 24 h of exposure [6, 28] and 93% of cows interacted with the brush within the first 7 days after installation [28]. Interestingly, our average time to the first contact were consistently smaller than those described for adult cows. One potential explanation may relate to pen size, as it has been suggested that adult cows reduce the usage of mechanical brushes when food is located far from this type of devices [4]. Another possible reason could be the exploratory behavior of young animals or to the need of young calves for maternal grooming [13].

## Conclusions

Our findings suggest that providing an automated brush to recently weaned calves housed in groups reduced the not-active time, while increasing eating time. No effect was evident on weight gain and presentation of diseases. Further research is encouraged to assess health and behavioral effects of enriched environments on weaned dairy calves.

## Methods

This study was conducted in an organic certified calf yard, located in Northern Colorado. Weaned calves were managed during the study period according to the guidelines set by the Institutional Animal Care and Use Committee of Colorado State University (Protocol ID: 17-7236A). Additionally, all the procedures were in accordance with farm SOP and management.

### Animals, housing and feeding

A total of 162 recently weaned Holstein heifer calves were enrolled in the study. Average (SD) age at enrollment was 94 (±7) d. Data were collected from October 29th, 2017 to December 29th, 2017 and each cohort of weaned heifers was followed for a period of 20 days to evaluate the effect of an automated grooming brush available in their group pens on behavior and health. Once the experiment concluded, calves remained in the rearing facility, following the routine procedures stablished by the farm.

Pre-weaning management and housing of calves in this operation were previously described [29, 30]. Briefly, after receiving colostrum, calves were transferred to the rearing facility during the first day of life. Heifer calves were housed in individual polyethylene hutches (Agri-Plastics, Stoney Creek, ON, Canada) with straw bedding and a wire gate enclosure; calves had visual but no physical contact with other animals until weaning.

Pasteurized milk was provided 3 times a day in 2.8 L plastic bottles (E-Z Nurse™). A stepdown weaning process started at 73 ± 7 d of age and was completed in a 3 weeks period. In the beginning of each week one milk feeding was suspended and calves were closely monitored for disease, before being transferred to group pens.

Healthy weaned calves were moved from individual hutches to group housing based on space availability, consumption of calf starter (1.8 kg to 2.2 kg per day), weight (minimum 72 kg) and age. Animals were housed in groups of 20 to 22 calves in pens located in the same facility.

Nine pens conformed the group housing area (128 m × 24 m). A solid wall in the back and a ceiling covering the bedding area offered shelter. Clean and dry straw bedding was provided for each pen before calf arrival and new bedding was given as needed.

Eight pens (24 m × 16 m) were used, including a covered area (7 m × 16 m) and a feeding area (16 m × 3 m) in each pen. A 9th pen was used for weak calf monitoring. Calves were housed in the described facility for 3 weeks before being moved to a different location. One automated grooming brush was installed in every other pen (*n* = 4).

Feed was delivered in a feed bunk lane at 07:00 h and 17:00 h. The daily ration consisted of increasing amounts of 3.6 to 4.5 kg of calf mix, corresponding to 75% calf starter and 25% TMR (53% Moisture, 47% dry matter, 16% crude protein) per calf. Two water troughs were available per pen.

Animal husbandry and treatments were provided as specified in the farm protocols. Personnel evaluating calf health was unaware of the objectives of the current study.

### Experimental design and treatments groups

For the experimental design and acknowledging the limitation for pen replicates, calves randomly nested in 2 factors. First, in the pen by EG date; second, in the TG. One CON and one AB group were enrolled simultaneously per each EG. Calf was used as experimental unit and EG was considered a pseudo-replicate.

Enrollment group 1 was enrolled in October 29, 2017 (CON = 22, AB = 22). Subsequent EG were enrolled in November 8 (CON = 19, AB = 20), November 17 (CON = 20, AB = 20), and November 27 (CON = 20, AB = 19). The enrollment was performed at the day when calves were fully weaned, and heifers remained in the individual hutches for 10 ± 3 days after complete weaning. Behavioral and health records were analyzed starting the day of transfer to group housing.

Calves were subject to a clinical evaluation at enrollment, following the farm SOP only healthy animals were included in the study. Each calf was weighted using a mobile crate scale (LFT-700S, W-W Paul Scale, Duncan, OK) at enrolment and at the end of the study period.

Transferring to group pens was performed by trained personnel under the supervision of the authors. Study calves were monitored in group housing for 20 days. Treatment and control groups experienced the same handling and housing conditions, except for the presence of the automated grooming brush. Clinical disease events and treatments administered to heifer calves by farm personnel were collected from farm records.

#### Automated grooming brush

One automated grooming brush was installed in 4 pens (Comfort Brush™ for calves, Future Cow^R^, Longwood, FL). Brushes were 60.9 cm long and 45 cm diameter, with 360° of horizontal movement and 45° of vertical swing. A motion sensor automatically activated the rotation of the brush that ramped up slowly. Brushes were located under the covered area in the middle of the pen, 60 cm above the ground with the motion sensor facing the bedding area. Brushes were placed in every other pen, with 4 CON and 4 AB pens separated by a fence.

### Data collection

#### Accelerometer sensors

Previous to calf enrolment, a small-scale trial with weaned heifer calves was completed to determine adequate ear placement and to envision possible problems associated to readings and ear infection or inflammation. Some problems following ear tag application, such as infection and subsequent increased ear movement, were detected. To assess this problem, we removed the farm ID tag from the left ear providing the opportunity to place the sensor in the middle of the ear, as it is recommended by previous reports [18] and the manufacturer. In addition, this procedure reduced the potential for skin damage. Consequently, at enrolment the left ear was examined for irritation or infection and the farm identification (ID) ear tag was temporarily removed. After disinfection, a 3-D accelerometer sensor (Cow Manager SensOor, Agis, Harmelen, The Netherlands) was attached replacing the farm ID. Each 3-D accelerometer sensor had an individual number that was linked to a specific calf in the system’s software. A solar power antenna was installed in the group housing area to maintain continuous communication between sensors and the master laptop located in the rearing facility.

The system provided exclusive measurements in minutes per hour for active, high active, not-active, eating and ruminating time [18, 19]. When the monitoring period was completed, the 3-D accelerometer sensors were removed.

#### Recording camera system

One camera programmed for continuous recording was installed to register the interaction of AB calves with the automated grooming brush per AB pen (1080p HD surveillance, model: dvr4–4575, Swamm, Santa Fe Springs, CA).

The camera positioning allowed the recording of the complete automated brush and the correct identification of the calf ID using or close to the brush. Continuous recording of 24 h were collected for 7 days to determine a first interaction. The first interaction with the brush was defined as any physical contact with the device, requiring that the calf was aware of the brush. In this first contact, the total time of use and the anatomic area of contact was recorded.

#### Health status

Caretakers followed the farm SOP for treatment of sick animals. Every morning, all pens were walked and calves with any sign of depression, mucus, cough, or diarrhea were examined. Rectal temperature was measured [31] and support therapy was administered if necessary. Calves that did not show improvement were moved to large collective hutches, conditioned in an isolation area that housed groups of 3 to 4 calves. All heifer calves that left the group housing due to disease had the 3-D accelerometer sensor removed. The animals were delinked from the system and considered out of the study.

### Statistical analysis

Data were analyzed using SAS statistical software (9.4, SAS Institute Inc., Cary, NC). Least square means were calculated for initial (weaning) and final weight, weight gain and ADG using PROC GLM. Logistic regression (PROC LOGISTIC) was used for the analysis of binary outcome variables, including detection of sick calves and removal from the group housing due to disease. Models included treatment group (CON; AB) and enrollment group by date (EG = 1 to 4). Kaplan Meier survival analyses (PROC LIFETEST) were performed to determine group differences in the time to the occurrence of the first disease and medical treatment administration. Wilcoxon test was used to determine statistical differences.

The time spent by calves as not-active, active, high active, eating and ruminating during the 20 days in study was analyzed aggregating the data by day in the study (1 to 20). Least square means were estimated for each of these response variables using repeated measures ANOVA for nested factors (PROC MIXED). Subjects were nested in EG and TG. The model included EG (nested in TG), TG, day in study, and the interaction between TG and day in study. Statistical significance was stablished at *P* ≤ 0.05. Data from video recording for the first interaction were summarized as the average time to the first use by each calf.

## Data Availability

The datasets generated and/or analyzed during the current study are not publicly available due to the extension of the behavioral records but are available from the corresponding author on reasonable request.

## Abbreviations

AB: Automated brush
ADG: Average daily gain
CI: Confidence interval
CON: Control group
EG: Enrollment group
ID: Identification
OR: Odds ratio
SOP: Standard operating procedure
TG: Treatment group
